# Burnout Levels and Associated Factors Among Newly Graduated Nurses: A Systematic Review and Meta‐Analysis

**DOI:** 10.1155/jonm/4994782

**Published:** 2026-08-17

**Authors:** Yao Zhou, Zhexi Ying, Akiko Kondo

**Affiliations:** ^1^ International Nursing Development, Graduate School of Health Care Sciences, Institute of Science Tokyo, 1-5-45 Yushima, Bunkyo-ku Tokyo, 113-8510, Japan, tokyo.ac.jp

**Keywords:** burnout, meta-analysis, newly graduated nurses, systematic review, transition

## Abstract

**Background:**

The transition into the workplace exposes newly graduated nurses to significant stressors and challenges that enhance their vulnerability to burnout. However, evidence on the level of burnout and its related factors in newly graduated nurses remains limited.

**Objectives:**

To determine the prevalence and severity of burnout among newly graduated nurses, identify its associated factors, and examine differences across geographic regions and before versus during the COVID‐19 pandemic.

**Methods:**

A systematic search of PubMed, CINAHL, MEDLINE, Web of Science, Japan Medical Abstracts Society, and China National Knowledge Infrastructure databases was performed from inception to July 2024. Pooled mean scores for three subscales of the 22‐item Maslach Burnout Inventory were calculated using R software (Version 4.4.1) to assess burnout levels. Publication bias tests, sensitivity analyses, and subgroup analyses were performed. Burnout prevalence and associated factors were summarized using narrative synthesis.

**Results:**

Forty‐five studies involving 18,203 newly graduated nurses were included. The reported rates of high‐level burnout ranged widely from 1.0% to 78.3%. The pooled mean scores were 28.39 (95% CI: 26.17–30.61) for Emotional Exhaustion, 11.48 (95% CI: 9.40–13.55) for Depersonalization, and 24.99 (95% CI: 21.22–28.76) for Personal Accomplishment. Subgroup analyses showed that newly graduated nurses in Asia had higher depersonalization (12.48 vs. 8.24) and lower personal accomplishment scores (22.34 vs. 32.99) than non‐Asian counterparts (both *p* < 0.01). Factors associated with burnout included personal traits and competencies (such as coping strategies and psychological capital), work‐related factors (such as work environment and workplace bullying and incivility), and other factors such as social support; however, evidence regarding sociodemographic factors was mixed.

**Conclusions:**

Newly graduated nurses experienced severe burnout, especially in Asia. Factors related to burnout among newly graduated nurses are multifaceted, highlighting the need for nursing managers to prioritize targeted strategies to facilitate their transition to professional roles. Structured orientation, effective preceptorship, supportive work environments, and accessible psychological support may help reduce burnout among newly graduated nurses.

## 1. Introduction

Burnout is defined as a syndrome caused by chronic occupational stress and manifested by Emotional Exhaustion (EE), Depersonalization (DP), and reduced Personal Accomplishment (PA) [1]. Burnout has become a critical issue across the healthcare system [2]. Notably, nurses exhibit higher burnout rates than other healthcare professionals [3]. Previous meta‐analyses reported that the prevalence of high‐level burnout in the nurse population was 11.2%, compared to 6% in the physician population [4, 5]. Burnout can adversely impact nurses’ health, work performance, and turnover, leading to compromised care quality and patient safety [2, 6]. According to a national survey in the United States, burnout accounted for 31.5% of nurses who left their positions [7]. Ultimately, the nursing burnout issue may impact healthcare systems on a national or regional scale [2].

Among them, the transition shock from school to clinical practice makes newly graduated nurses particularly vulnerable to mental health issues [8]. Even after a few years of training, novice nurses may still feel underprepared and stressed [9]. Their large‐scale longitudinal study found that about one‐fifth of newly graduated nurses encounter very high levels of burnout within the initial 3 years of practice, with changes in burnout often corresponding to changes in their intention to leave. Compared to experienced colleagues, newly graduated nurses are more susceptible to burnout and show a higher intention to leave their jobs [10]. Given the ongoing global nursing shortage, the capacity to recruit and retain newly graduated nurses is critical to sustaining a stable healthcare workforce [11]. Interventions targeting burnout may be valuable strategies to address the retention of new nurses [8].

The COVID‐19 pandemic has further emphasized the urgency of strengthening the health workforce worldwide. During this period, nurses have faced increased job demands and workloads, insufficient staffing and resources, inadequate pandemic‐specific training, and fear of infection, which combined to exacerbate their job stress and lead to increased burnout [12]. Studies reported that up to 80.2% of nurses encountered burnout during the COVID‐19 pandemic [3]. The challenges arising from this healthcare crisis are particularly pronounced among novice nurses. With orientation programs shortened or even canceled during this period, new nurses entering the practice felt inadequately prepared for their work and experienced considerable stress [13]. This public health emergency left them with insufficient time to adapt to their roles and develop professional confidence, resulting in both physical and psychological exhaustion [14]. Most previous reviews have focused on the general nursing workforce during the pandemic, thereby neglecting the particular vulnerabilities of newly graduated nurses. In addition, previous studies have identified regional variations in burnout levels among nurses [15, 16]. A meta‐analysis of oncology nurses revealed that nurses in Asia scored highest on the dimensions of EE and DP, while European nurses scored lowest on the dimension of reduced PA, indicating significant regional differences in burnout severity [15]. However, whether similar regional differences exist among newly graduated nurses remains unclear.

To date, only one scoping review specifically summarized burnout among newly graduated nurses [17]. According to this review, the prevalence of burnout among newly graduated nurses (12.3%–46%) was similar to that among other healthcare professionals. However, this finding was based on only three original studies and therefore cannot determine the overall severity of burnout in this population. Furthermore, the following limitations prevent it from providing sufficient evidence to guide policy or practice: a small number of included studies, restriction to English‐language literature, inclusion of studies published only between 2011 and 2021, and the use of narrative synthesis methodology. Hence, there remains a need for a more comprehensive and systematic approach to evaluate the level of burnout and identify its associated factors in newly graduated nurses, while also examining the impact of the pandemic and regional variations. Addressing these knowledge gaps will help build a stronger evidence base for developing targeted interventions and workforce policies. Therefore, this review aims to address the following questions: (1) What is the prevalence and severity of burnout among newly graduated nurses? (2) Do the COVID‐19 pandemic and the geographic regions affect burnout levels among newly graduated nurses? (3) What factors are associated with burnout among newly graduated nurses?

## 2. Methods

This review was implemented in accordance with the methodological framework of the Joanna Briggs Institute (JBI) guidelines for systematic reviews [18], and the protocol was registered beforehand in PROSPERO (ID: CRD42024554981). Reporting procedures followed the Preferred Reporting Items for Systematic Reviews and Meta‐Analyses (PRISMA) 2020 statement [19].

### 2.1. Eligibility Criteria

The inclusion criteria were as follows: (1) participants or subgroup participants were newly graduated nurses; (2) studies investigated the prevalence or related factors of burnout using any validated measurement or investigated scores on the three dimensions of burnout using the 22‐item Maslach Burnout Inventory–Health Services Survey (MBI–HSS) scale; (3) observational studies, including cross‐sectional and cohort studies; and (4) peer‐reviewed original studies published in English, Chinese, or Japanese. There were no restrictions on publication year. According to Benner’s From Novice to Expert model, nurses generally require 3 years of clinical experience to progress from the novice to the competent stage of professional practice [20]. Therefore, this review defined newly graduated nurses as registered nurses who have up to 3 years of professional experience [21], which also ensured sufficient study numbers for the meta‐analysis.

The exclusion criteria were (1) unable to extract separate data for newly graduated nurses; (2) gray literature, perspective, opinion, review articles, conference abstracts, commentaries, dissertations, and theses; or (3) poor‐quality studies.

### 2.2. Search Strategy

We adopted a three‐phase search strategy, starting with an initial search on PubMed to identify suitable keywords. The six electronic databases searched were PubMed, CINAHL, MEDLINE, Web of Science, Japan Medical Abstracts Society, and China National Knowledge Infrastructure. The combination of keywords was: (“newly graduate∗ nurse∗” OR “new graduate∗ nurse∗” OR “newly qualified nurse∗” OR “novice nurse∗” OR “fresh nurse∗” OR “first year nurse∗” OR “newly registered nurse∗” OR “new staff nurse∗” OR “newly licensed nurse∗” OR “new nurse∗” OR “neophyte nurse∗”) AND “Burnout”. The search timeframe was from the database inception to July 2024. Details of the search strategies and preliminary results are provided in Supporting Table S1. Finally, reference lists of included studies were screened to locate additional eligible studies.

### 2.3. Study Selection

Records retrieved from each database were imported into Zotero 6.0, and duplicate records were deleted. Titles and abstracts, then full‐text articles, were assessed by two independent reviewers (Yao Zhou and Zhexi Ying) using the predefined inclusion criteria. In cases where library access did not provide the full text, we requested the complete version from the study authors using ResearchGate or email. Two independent reviewers (Yao Zhou and Zhexi Ying) proficient in English, Chinese, and Japanese read the original text directly rather than translations to avoid potential misinterpretations. Any disagreements between the two reviewers were settled through discussion or consultation with the third reviewer (Akiko Kondo), who is a native Japanese speaker with advanced English proficiency.

### 2.4. Quality Assessment

Two reviewers (Yao Zhou and Zhexi Ying) independently assessed the methodological quality of studies using the JBI critical appraisal tool 2020 (including a checklist for analytical cross‐sectional studies and a checklist for cohort studies) [18]. The response options for each checklist item are “yes,” “no,” “unclear,” or “not applicable.” Based on the proportion of “yes” responses, the quality of studies was categorized as good (≥ 80%), moderate (50%–79%), or poor (< 50%) [8]. To enhance the reliability of the review, studies rated as poor quality were excluded [18]. Any disagreements were addressed by discussion or consultation with the third reviewer (Akiko Kondo).

### 2.5. Data Extraction

Two independent reviewers (Yao Zhou and Zhexi Ying) read the included studies and extracted the following data: author(s), publication year, study design, country, sample size, data collection time, burnout measurements, and burnout outcomes (including prevalence, burnout scores, and associated factors). All extracted data were entered into data sheets (Microsoft Excel) for further synthesis. Data from cross‐sectional studies and first‐time or baseline data on newly graduated nurses’ burnout levels from longitudinal studies were extracted. Disagreements were resolved through discussion or by the third reviewer (Akiko Kondo).

### 2.6. Data Synthesis

The meta‐analysis was performed using R software (Version 4.4.1). Only studies that provided mean scores and standard deviations for the three subscales of the 22‐item MBI–HSS were included for meta‐analysis. Burnout severity was quantitatively evaluated by calculating pooled means and their 95% confidence intervals (95% CI) from the included studies. Because all studies reported outcomes using the same scale and range, means were directly pooled rather than converted into standardized mean differences to ensure methodological consistency across both the meta‐analysis and subgroup analyses [22]. Heterogeneity was examined using the *I* ^2^ statistic. Given the significant heterogeneity observed across the three dimensions (*I* ^2^ ≥ 50%), random‐effects models were fitted using restricted maximum likelihood (REML) estimation, with Knapp–Hartung adjustment. Publication bias was evaluated using Egger’s test. To evaluate the robustness and reliability of the findings, a sensitivity analysis was conducted using a leave‐one‐out method. Subgroup analyses were conducted to examine the sources of heterogeneity, including differences by data collection timing (before vs. during the COVID‐19 pandemic), geographic region (categorized as Asia vs. non‐Asia due to data availability), and study quality (moderate vs. good), and to assess their effects on burnout levels. The statistical significance of subgroup differences was assessed using the *Q* test based on the chi‐square distribution, with a *p*‐value less than 0.05 regarded as significant. As the World Health Organization declared COVID‐19 a pandemic on March 11, 2020, data collected before this date were classified as “before the pandemic,” whereas data collected afterward were classified as “during the pandemic” [23]. One study had a data collection period that spanned both periods without providing separate data [24]; therefore, it was excluded from the subgroup analysis of data collection timing. The subgroup analyses included 12 studies, which met the commonly accepted empirical criterion (≥ 10 studies) for conducting subgroup analyses [22]. To further explore potential sources of heterogeneity, we conducted exploratory meta‐regression analyses. We examined publication year, sample size, proportion of female participants, and mean age as continuous study‐level moderator variables. Meta‐regression analyses were restricted to studies reporting the corresponding covariate; therefore, the number of included studies varied across models. The highly heterogeneous nature of burnout prevalence and associated factors in the included studies prevented a meta‐analysis, so we used a narrative synthesis. Findings on potential factors associated with burnout were extracted from the included studies. We extracted studies reporting positive, negative, and nonsignificant associations for each reported factor and conducted a narrative synthesis based on the direction and consistency of the findings.

## 3. Results

### 3.1. Search Results

**FIGURE 1:**
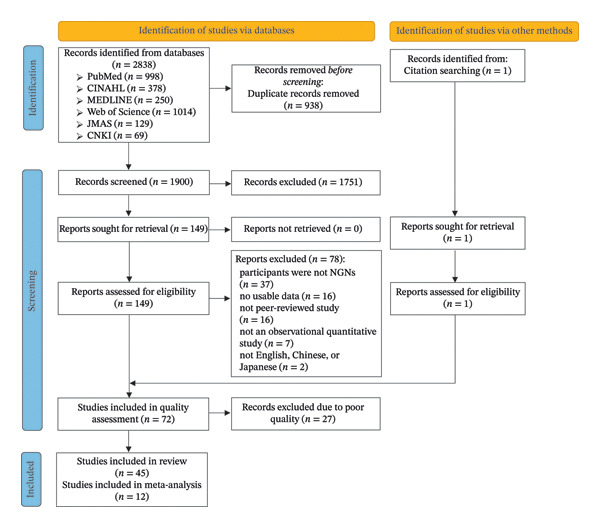
PRISMA 2020 flow diagram.

We initially identified 2838 studies. After removing duplicates and screening the titles and abstracts of the remaining studies, we excluded 2689 studies. The remaining 149 studies, together with one study identified through citation searching, underwent full‐text assessment, with 78 excluded for not meeting the eligibility criteria and 27 excluded for poor quality. Finally, 45 studies were included in this review, of which 12 met the inclusion criteria for meta‐analysis. Figure 1 illustrates the flow of the study selection process.

### 3.2. Study Characteristics

Forty‐five studies involving 18,203 newly graduated nurses were included in this review, of which 39 were cross‐sectional and six were longitudinal. The included studies involved 10 different countries, with the majority of studies conducted in China (*n* = 23), followed by Canada (*n* = 7), Japan (*n* = 4), South Korea (*n* = 4), the United States (*n* = 2), Australia (*n* = 1), Poland (*n* = 1), Spain (*n* = 1), Sweden (*n* = 1), and the United Kingdom (*n* = 1). Thirty‐one studies were published in English, 12 in Chinese, and two in Japanese. Various burnout measurement tools were used in the included studies, with 36 (80.0%) using the Maslach Burnout Inventory (including the MBI–HSS and Maslach Burnout Inventory–General Survey, MBI–GS). Publication years ranged from 2006 to 2024, with 27 studies (60%) published within the last five years (2020–2024). The detailed characteristics of the included studies are described in Table 1. The quality appraisal of studies is presented in Supporting Tables S2 and S3.

**TABLE 1:** Characteristics of included studies (*N* = 45).

Author(s), year	Study design	Country/language	Sample	Data collection time	Measurements	Burnout outcomes			Quality score/level
						Prevalence	Scores (mean ± SD)	Associated factors	
Cao et al., 2021 [43]	Cross‐sectional	China/English	393 NGNs with ≤ 1 year experience	July to August 2020	ProQOL	38.2% moderate burnout	21.30 ± 5.14	Educational level, coping strategies, empathy, resilience, compassion satisfaction, occupational stress, transition shock	75.0%/moderate
Catarelli et al., 2023 [64]	Cross‐sectional	United States/English	43 NGNs with < 1 year experience	July to July 2021	CBI	NR	58.5 ± 19	Job satisfaction	50.0%/moderate
Chang and Kim, 2022 [55]	Longitudinal	South Korea/English	212 NGNs with < 1 year experience	September to October 2020	BM	NR	Total average 3.43 ± 0.59	workplace violence	54.6%/moderate
Chen, 2012 [79]	Longitudinal	China/Chinese	49 NGNs with < 3 years’ experience	July 2011 (baseline)	MBI–HSS	6.1% high burnout	NR	NR	63.6%/moderate
Dwyer et al., 2019 [41]	Cross‐sectional	United States/English	136 NGNs with 6 months to 3 years’ experience	January to February 2016	MBI–GS	51.5% high burnout	EE average 3.32 ± 1.46	Age, psychological capital, leadership, structural empowerment	87.5%/good
Gong et al., 2022 [46]	Cross‐sectional	China/English	315 NGNs with < 1 year experience	March to April 2021	ProQOL	43.2% moderate burnout	24.39 ± 5.20	Empathy, work environment, social support	100%/good
Guo et al., 2022 [54]	Cross‐sectional	China/Chinese	492 NGNs with 6–36 months’ experience	October to December 2019	MBI–GS	NR	Total average 2.12 ± 0.97, EE average 2.51 ± 1.34, DP average 1.64 ± 1.25, PA average 2.08 ± 1.36	Psychological empowerment	87.5%/good
Hu and Xiao, 2022 [63]	Cross‐sectional	China/Chinese	172 NGNs with 10–12 months’ experience	June to August 2019	MBI–HSS	NR	NR	Transition shock	62.5%/moderate
Kaneko, 2012 [56]	Cross‐sectional	Japan/Japanese	293 NGNs with < 3 years’ experience	October to November 2010	MBI–HSS	NR	NR	Coping strategies, stress	62.5%/moderate
Kim and Yang, 2023 [57]	Cross‐sectional	South Korea/English	210 NGNs with < 1 year experience	2 May to 30 June 2023	BM	NR	Total average 2.65 ± 0.60	workplace relationship	62.5%/moderate
Kim et al., 2019 [30]	Cross‐sectional	South Korea/English	124 NGNs with ≤ 1.9 years’ experience	July to September 2018	MBI–HSS	NR	EE 35.30 ± 11.18, DP 14.85 ± 6.62, PA 19.35 ± 7.86	NR	62.5%/moderate
Kim et al., 2022 [45]	Cross‐sectional	South Korea/English	325 NGNs with < 3 years’ experience	February to March 2021	MBI–HSS	NR	< 1 year (*n* = 130): 63.90 ± 18.53, 1–2 years (*n* = 106): 67.96 ± 18.14	Work tenure, social group membership	62.5%/moderate
Laschinger, 2011 [65]	Cross‐sectional	Canada/English	342 NGNs with < 2 years’ experience	July to October 2010	MBI–GS	NR	1 year (*n* = 153): EE average 2.87 ± 1.66, cynicism average 1.82 ± 1.40, personal efficacy average 4.54 ± 0.98; 2 years (*n* = 189): EE average 2.92 ± 1.38, cynicism average 1.67 ± 1.30, personal efficacy average 4.68 ± 0.88	Job satisfaction	50.0%/moderate
Laschinger and Grau, 2011 [50]	Cross‐sectional	Canada/English	165 NGNs with 1–12 months’ experience	July to October 2010	MBI–GS	39.6% high EE, 19.5% high DP	EE average 2.82 ± 1.64, cynicism average 1.84 ± 1.41	Psychological capital, person‐job fit, workplace bullying	50.0%/moderate
Laschinger and Read, 2016 [59]	Cross‐sectional	Canada/English	993 NGNs	November 2012 to March 2013	MBI–GS	NR	EE average 3.23 ± 1.47	Person‐job fit, leadership, workplace incivility	50.0%/moderate
Laschinger et al., 2010 [58]	Cross‐sectional	Canada/English	247 NGNs with < 2 years’ experience	2006	MBI–GS	NR	EE average 3.43 ± 1.38	Work environment, workplace incivility, structural empowerment	50.0%/moderate
Laschinger et al., 2012 [60]	Cross‐sectional	Canada/English	342 NGNs with < 2 years’ experience	July to October 2010	MBI–GS	NR	EE average 2.90 ± 1.51, cynicism average 1.74 ± 1.34	Leadership, structural empowerment	50.0%/moderate
Laschinger et al., 2012 [61]	Cross‐sectional	Canada/English	342 NGNs with ≤ 2 years’ experience	July to October 2010	MBI–GS	NR	EE average 2.90 ± 1.51	Leadership, workplace bullying, job satisfaction	62.5%/moderate
Laschinger et al., 2015 [49]	Cross‐sectional	Canada/English	1009 NGNs with < 3 years’ experience	NR	MBI–GS	NR	EE average 3.24 ± 1.48, cynicism average 1.60 ± 1.56	Person‐job fit, leadership, self‐efficacy	50.0%/moderate
Ma et al. 2020 [27]	Cross‐sectional	China/English	366 NGNs with ≤ 3 years’ experience	January to February 2020	MBI–HSS	52.5% high burnout, 60.9% high EE, 74.0% high DP, 95.4% high PA	Total 62.25 ± 14.19, EE 29.31 ± 8.81, DP 12.85 ± 4.68, PA 20.09 ± 7.76	Mobile phone addiction	62.5%/moderate
Ma et al., 2021 [28]	Cross‐sectional	China/English	220 NGNs with < 3 years’ experience	June to July 2020	MBI–GS	6.8% high EE, 1.8% high DP, 15.0% high PA	EE 15.72 ± 5.07, DP 9.48 ± 4.04, PA 17.98 ± 9.59	Age, marital status, work procrastination, mobile phone addiction	62.5%/moderate
Ma et al., 2022 [29]	Cross‐sectional	China/Chinese	385 NGNs with ≤ 3 years’ experience	December 2019 to March 2020	MBI–HSS	65.2% high EE, 81.3% high DP, 95.1% low PA	EE 29.32 ± 8.90, DP 12.86 ± 4.86, PA 20.12 ± 7.82	NR	62.5%/moderate
Molina‐Mula et al. 2021 [31]	Cross‐sectional	Spain/English	77 NGNs with < 3 years’ experience	February to March 2021	MBI–HSS	NR	EE 28.81 ± 11.99, DP 9.0 ± 6.66, PA 28.51 ± 6.82	NR	75.0%/moderate
Nakazawa et al., 2024 [44]	Cross‐sectional	Japan/Japanese	539 NGNs	January to March 2019	MBI–HSS	NR	13.51 ± 2.42	Educational level, coping strategies, psychological capital, work engagement, quality of work life, job satisfaction, social support	62.5%/moderate
Peng et al., 2015 [62]	Cross‐sectional	China/Chinese	630 NGNs with < 1 year experience	November to December 2014	MBI–HSS	NR	Total 54.75 ± 5.91, EE 24.17 ± 7.04, DP 11.03 ± 3.85, PA 25.63 ± 4.89	Work environment	50.0%/moderate
Ren and Kim, 2023 [21]	Cross‐sectional	China/English	405 NGNs with 6 months to 3 years’ experience	November 2020 to February 2021	MBI–GS	NR	30.9 ± 3.21	Psychological empowerment, workplace bullying	75.0%/moderate
Rudman et al., 2020 [80]	Longitudinal	Sweden/English	2423 NGNs	2002, 2004, and 2006 (baseline)	OLBI	5.2% high burnout	NR	NR	72.7%/moderate
Serafin et al., 2022 [25]	Cross‐sectional	Poland/English	212 NGNs with 6 months–36 months’ experience, Group I: 120 NGNs, Group II: 92 NGNs	Group I: October to December 2019, Group II: September 2020 to October 2021	OLBI	78.3% high burnout	Group I: Exhaustion 20.68 ± 4.47, Disengagement 19.77 ± 4.09; Group II: Exhaustion 23.74 ± 3.63, Disengagement 21.30 ± 3.60	Workplace bullying, worked during the COVID‐19 pandemic	50.0%/moderate
Shi et al., 2018 [51]	Cross‐sectional	China/English	696 NGNs with < 3 years’ experience	May 2016	MBI–GS	NR	Total average 3.19 ± 0.84	Resilience, workplace incivility	50.0%/moderate
Sun et al., 2021 [47]	Cross‐sectional	China/English	245 NGNs	June to July 2021	MBI–HSS	NR	Total 42.32 ± 16.18, EE 18.03 ± 6.47, DP 15.69 ± 5.79, PA 11.56 ± 6.52	Quality of work life, professional identity, work engagement	62.5%/moderate
Suzuki et al., 2006 [67]	Longitudinal	Japan/English	923 NGNs	June 2003 (baseline)	MBI–HSS	NR	Total average 12.2 ± 2.3, EE average 3.7 ± 1.1, DP average 1.5 ± 1.1, PA average 3.0 ± 1.1	Social support	72.7%/moderate
Suzuki et al., 2008 [66]	Longitudinal	Japan/English	923 NGNs	June 2003 (baseline)	MBI–HSS	NR	Total average 12.2 ± 2.3, EE average 3.7 ± 1.1, DP average 1.5 ± 1.1, PA average 3.0 ± 1.1	Job satisfaction, social support	81.8%/moderate
Tan et al., 2023 [32]	Cross‐sectional	China/Chinese	582 NGNs with ≤ 3 years’ experience	August to October 2021	MBI–HSS	NR	Total 61.74 ± 14.32, EE 26.11 ± 8.79, DP 9.76 ± 5.64, PA 25.37 ± 8.41	Mindfulness, emotional intelligence	62.5%/moderate
Theodosius et al., 2020 [33]	Cross‐sectional	United Kingdom/English	58 NGNs with 6 months to 2 years’ experience	November 2017 to April 2018	MBI–HSS	NR	EE 24.45 ± 12.28, DP 5.97 ± 5.19, PA 34.78 ± 8.20	NR	62.5%/moderate
Wang et al., 2023 [42]	Cross‐sectional	China/English	506 NGNs with ≤ 2 years’ experience	August 2021 to July 2022	ProQOL	NR	21.87 ± 6.46	Gender, work tenure, coping strategies, death competence	75.0%/moderate
West et al., 2007 [34]	Longitudinal	Australia/English	37 NGNs	NR	MBI–HSS	NR	EE 26.8 ± 10.5, DP 10.1 ± 7.1, PA 35.8 ± 5.7	Coping strategies, job satisfaction	54.6%/moderate
Xie et al., 2020 [35]	Cross‐sectional	China/English	2071 NGNs with < 3 years’ experience	January to July 2019	MBI–HSS	NR	EE 23.84 ± 8.33, DP 7.51 ± 6.02, PA 28.64 ± 7.70	Gender, work tenure, hospital level, core competence, self‐efficacy, professional values, job satisfaction	87.5%/good
Xie et al., 2022 [52]	Cross‐sectional	China/Chinese	128 NGNs with < 1 year experience	January 2019 to January 2020	MBI–GS	NR	Total 58.46 ± 5.21, EE 16.12 ± 2.16, DP 16.56 ± 2.45, PA 27.31 ± 3.42	Occupational stress, resilience, self‐efficacy	62.5%/moderate
Xu et al., 2015 [37]	Cross‐sectional	China/Chinese	50 NGNs	April to June 2013	MBI–HSS	NR	EE 28.51 ± 6.82, DP 10.66 ± 7.13, PA 27.04 ± 8.25	NR	75.0%/moderate
Xu et al., 2022 [36]	Cross‐sectional	China/Chinese	246 NGNs with < 1 year experience	July to November 2020	MBI–HSS	NR	Total 66.23 ± 12.41, EE 30.20 ± 7.59, DP 16.19 ± 3.36, PA 19.84 ± 4.24	Age, gender, educational level, marital status, internship time, night shifts, occupational stress, role switching pressure	100%/good
Zeng and Cui, 2014 [53]	Cross‐sectional	China/Chinese	480 NGNs with < 1 year experience	November to December 2013	MBI–HSS	NR	Total average 3.89 ± 0.86, EE average 4.01 ± 0.75, DP average 3.01 ± 0.90, PA average 2.41 ± 0.79	Resilience	62.5%/moderate
Zeng et al., 2023 [26]	Cross‐sectional	China/English	520 NGNs with 6 months to 3 years’ experience	June 2021 to October 2021	ProQOL	1.0% high burnout	27.94 ± 5.04	Marital status, sleep time, night shifts, workload, workplace violence, job satisfaction	62.5%/moderate
Zhai et al., 2023 [48]	Cross‐sectional	China/Chinese	384 NGNs with ≤ 2 years’ experience	April to July 2021	MBI–GS	NR	Total 43.14 ± 16.12, EE 19.14 ± 6.76, DP 10.68 ± 5.67, PA 13.33 ± 8.36	Psychological capital, work engagement	62.5%/moderate
Zhao et al., 2023 [38]	Cross‐sectional	China/English	481 NGNs with ≤ 3 years’ experience	July 2021 to August 2022	MBI–HSS	NR	EE 33.48 ± 6.53, DP 16.07 ± 5.23, PA 20.75 ± 4.51	Educational level, work tenure, coping strategies, workplace relationship, stress, social support	100%/good
Zheng et al., 2023 [24]	Cross‐sectional	China/Chinese	184 NGNs	June 2019 to October 2021	MBI–HSS	NR	Total 57.66 ± 3.99, EE 26.04 ± 3.01, DP 11.45 ± 1.20, PA 20.18 ± 1.97	Age, educational level, income, work tenure, psychological capital, night shifts, stress	62.5%/moderate

*Note:* Numbers in brackets following the author(s) and year indicate the corresponding reference in the reference list.

Abbreviations: BM, burnout measure; CBI, Copenhagen Burnout Inventory; DP, depersonalization; EE, emotional exhaustion; MBI–GS, Maslach Burnout Inventory–General Survey; MBI–HSS, Maslach Burnout Inventory–Health Services Survey; NGNs, newly graduated nurses; NR, not reported; PA, personal accomplishment; ProQOL, Professional Quality of Life Scale; OLBI, Oldenburg Burnout Inventory.

### 3.3. Burnout Prevalence

Seven studies reported the prevalence of newly graduated nurses experiencing high levels of overall burnout, ranging from 1.0% to 78.3% [25, 26]. Three studies provided data on the prevalence of the three components of burnout, with 6.8%–65.2% of newly graduated nurses reported high levels of EE, 1.8%–81.3% with high levels of DP, and 15.0%–95.4% reported low PA [27–29].

### 3.4. Meta‐Analysis of Burnout Scores

**FIGURE 2:**
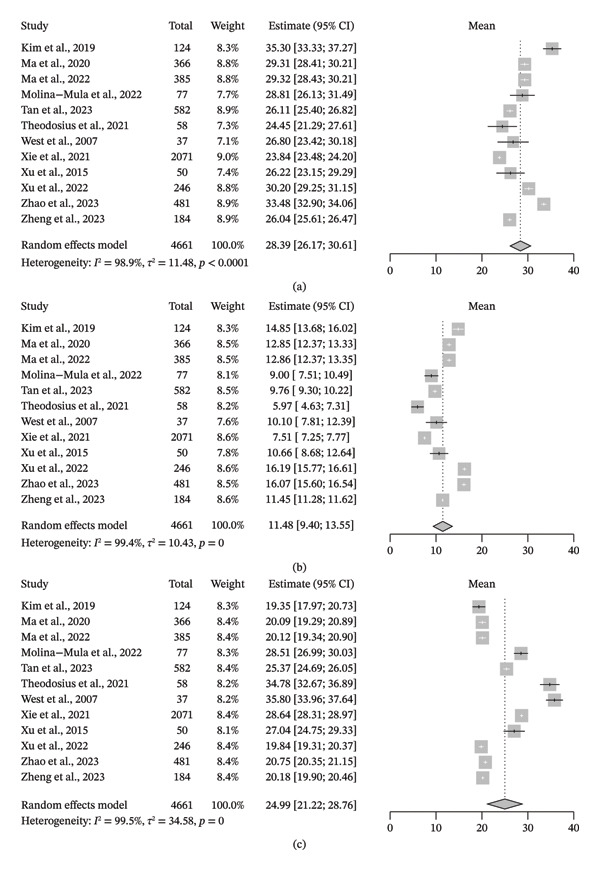
Forest plots of burnout scores by subscale: (a) Emotional Exhaustion; (b) Depersonalization; (c) Personal Accomplishment.

Twelve studies (*n* = 4661), published between 2007 and 2023, reported the means and standard deviations of the three subscale scores of the MBI–HSS [24, 27, 29–38]. The pooled mean score for EE was 28.39 (95% CI: 26.17–30.61), for DP was 11.48 (95% CI: 9.40–13.55), and for PA was 24.99 (95% CI: 21.22–28.76). These findings are presented in the forest plots in Figure 2. Burnout levels were assessed according to commonly used cutoff points in previous studies, with EE score ≥ 27, DP score ≥ 10, and PA score ≤ 33 corresponding to high burnout levels on each subscale [15, 39, 40]. The pooled mean scores indicated that newly graduated nurses generally experienced high EE and DP, accompanied by very low PA.

The heterogeneity (*I* ^2^) of EE, DP, and PA was 98.9%, 99.4%, and 99.5%, respectively. Egger’s test showed that the *p*‐values of EE, DP, and PA were 0.24, 0.65, and 0.54, respectively, indicating no statistically significant publication bias. Results from the sensitivity analysis showed no significant change in pooled estimates, suggesting the results were robust (see Supporting Figure S1).

The results of exploratory meta‐regression analyses showed that publication year, sample size, proportion of female participants, and mean age were not significantly associated with scores on the three subscales of burnout; furthermore, significant residual heterogeneity remained in all models. However, only seven studies provided data for the analysis of the proportion of female participants, and four studies provided data for the analysis of mean age. Therefore, the statistical power of these analyses was limited, and these findings should be interpreted cautiously. Detailed results are presented in Supporting Table S4.

### 3.5. Subgroup Analysis

Subgroup analysis was performed by different data collection timing, geographic region, and study quality. The pooled mean scores for all subgroups in the three subgroup analyses exceeded the cutoff points, indicating that newly graduated nurses within all subgroups exhibited high levels of burnout across the three dimensions. The studies that collected data during the COVID‐19 pandemic showed slightly higher EE scores (29.69 vs. 27.96), higher DP scores (12.79 vs. 10.70), and lower PA scores (23.58 vs. 26.51), but the differences were not statistically significant (*p* > 0.05). Newly graduated nurses in Asia showed significantly higher DP scores (12.48; 95% CI: 10.21–14.74) and significantly lower PA scores (22.34; 95% CI: 19.59–25.09) compared to non‐Asian counterparts (DP: 8.24; 95% CI: 2.92–13.57, PA: 32.99; 95% CI: 23.14–42.85, both *p* < 0.01). Good quality studies showed slightly higher EE (29.17 vs. 28.12) and DP scores (13.25 vs. 10.88), while PA scores were slightly lower (23.08 vs. 25.65), but none were statistically significant (*p* > 0.05). The results of the subgroup analysis are shown in Table 2.

**TABLE 2:** Results of the subgroup analysis.

Subgroups	Numbers of studies	Effect model	Emotional Exhaustion			Depersonalization			Personal Accomplishment		
			Mean scores (95% CI)	*I* ^2^	Test for subgroup differences	Mean scores (95% CI)	*I* ^2^	Test for subgroup differences	Mean scores (95% CI)	*I* ^2^	Test for subgroup differences
*Data collection timing*											
Before the COVID‐19 pandemic	7	Random	27.96 (24.33, 31.59)	98.1%	*x* ^2^ = 0.64, *p* = 0.42	10.70 (7.77, 13.64)	99.2%	*x* ^2^ = 0.83, *p* = 0.36	26.51 (20.05, 32.97)	99.4%	*x* ^2^ = 0.78, *p* = 0.38
During the COVID‐19 pandemic	4	Random	29.69 (24.71, 34.66)	98.8%		12.79 (6.57, 19.00)	99.5%		23.58 (17.14, 30.02)	98.8%	

*Geographic regions*											
Asia	9	Random	28.86 (25.99, 31.74)	99.2%	*x* ^2^ = 1.33, *p* = 0.25	12.48 (10.21, 14.74)	99.6%	*x* ^2^ = 7.17, *p* < 0.01	22.34 (19.59, 25.09)	99.6%	*x* ^2^ = 17.02, *p* < 0.001
Non‐Asia	3	Random	26.79 (21.27, 32.32)	53.1%		8.24 (2.92, 13.57)	85.3%		32.99 (23.14, 42.85)	95.4%	

*Study quality*											
Moderate	9	Random	28.12 (25.66, 30.57)	94.6%	*x* ^2^ = 0.12, *p* = 0.73	10.88 (8.89, 12.87)	96.5%	*x* ^2^ = 0.63, *p* = 0.43	25.65 (20.73, 30.56)	98.9%	*x* ^2^ = 0.53, *p* = 0.47
Good	3	Random	29.17 (16.98, 41.36)	99.8%		13.25 (0.89, 25.62)	99.9%		23.08 (11.06, 35.10)	99.8%	

*Note:* *I* ^2^, *I*‐square heterogeneity statistic.

Abbreviation: CI, confidence interval.

### 3.6. Associated Factors

Associated factors of burnout among newly graduated nurses were categorized into four categories: sociodemographic, personal traits and competence, work‐related, and other factors. The direction and consistency of the reported associations for each potential factor are summarized in Supporting Table S5.

#### 3.6.1. Sociodemographic Factors

Sociodemographic factors such as young age [24, 28, 36, 41], being female [35, 37, 42], and low income [24] were associated with higher burnout. Three studies showed a positive correlation between educational level and burnout [38, 43, 44]; two others reported the opposite [24, 36]. Two studies suggested higher burnout in unmarried newly graduated nurses [26, 28], while another reported greater risk among married newly graduated nurses [36]. Two studies linked longer work tenure to higher burnout [42, 45], while three found higher burnout with shorter work tenure [24, 36, 38].

#### 3.6.2. Personal Traits and Competence Factors

Nurses’ work competence [28, 32, 35, 42, 43, 46] and work engagement [35, 44, 47, 48] were negatively associated with burnout. Twelve studies indicated that psychological capital—such as positive states like self‐efficacy and resilience—was associated with lower levels of burnout [24, 32, 35, 41, 43, 44, 48–53]. Psychological empowerment [21, 54] and positive and active coping strategies [34, 38, 42–44, 55, 56] also protected newly graduated nurses from burnout.

#### 3.6.3. Work‐Related Factors

Ten studies indicated that a supportive work environment was negatively related to burnout [38, 41, 46, 49, 57–62]. Whereas workplace bullying and incivility had the opposite result [21, 25, 26, 50, 51, 55, 58, 59, 61]. Occupational stress [24, 36, 38, 43, 52, 56], transition shock [36, 43, 63], and working during the COVID‐19 pandemic [25] were associated with higher levels of burnout. Other work‐related factors such as hospital level [35], internship length [36], quality of work life [26, 44, 47, 49, 50, 59], structural empowerment [41, 58, 60], and job satisfaction [26, 34, 35, 43, 44, 61, 64–66] were negatively associated with burnout. Two studies identified more night shifts related to higher burnout [26, 36], while one found the opposite [24].

#### 3.6.4. Other Factors

Six studies mentioned that limited social support was linked to greater burnout, and support deficiency exists across sources such as peers, supervisors, mentors, and organizations [38, 44, 46, 63, 66, 67]. Mobile phone addiction [27, 28], absence of social group membership [45], and short sleep duration [26] were positively associated with burnout.

## 4. Discussion

This review investigated the prevalence, severity, and associated factors of burnout among newly graduated nurses and analyzed the impact of the COVID‐19 pandemic and geographic regions on burnout. To our knowledge, this study is the first systematic review and meta‐analysis of burnout among newly graduated nurses.

The prevalence of high‐level burnout among newly graduated nurses ranged from 1.0% to 78.3%, with high EE ranging from 6.8% to 65.2%, high DP from 1.8% to 81.3%, and low PA from 15.0% to 95.4%. The findings suggest substantial variation in burnout prevalence across studies, likely due to differences in survey timing, geographic regions, tools, and other factors. Differences in the theoretical basis of different scales also contribute to variations in prevalence rates [5]. Participants in different countries or health systems may respond differently to measurement items, and different language versions of the same scale may also lead to differences in scores [68].

Meta‐analysis of 12 studies showed that the pooled mean scores for EE, DP, and PA were 28.39 (95% CI: 26.17–30.61), 11.48 (95% CI: 9.40–13.55), and 24.99 (95% CI: 21.22–28.76), respectively. The pooled burnout scores of newly graduated nurses were above the high‐level threshold in all three dimensions, indicating an overall high level of burnout. Compared to burnout scores for senior nurses and those considered at high risk for burnout as emergency nurses or oncology nurses [15, 33, 39], newly graduated nurses had higher levels of burnout on all three dimensions. Although nurses are all at high risk for burnout [5], newly graduated nurses appear to be more vulnerable due to limited clinical competence, lack of social support, not yet adapted to the job, and inadequate coping strategies leading to severe burnout [8, 43]. Despite the theoretical foundation provided by nursing education, the disparity between theory and clinical practice often leaves novice nurses feeling inadequately prepared and lacking knowledge [69]. Compared to experienced nurses, they frequently lack confidence and security, making them more vulnerable when facing stress [69, 70].

Subgroup analysis indicated that burnout did not differ significantly between the pre‐pandemic and during the pandemic, aligning with the findings of a previous systematic review [71]. Although the pooled mean scores and 95% confidence intervals in our analysis indicated higher burnout during the pandemic, the small magnitude of difference suggests that the true effect was minimal and not clinically meaningful. The heterogeneity introduced by variations in study methodologies, healthcare settings, and local pandemic severity further diminishes the impact of minor differences. In routine medical practice, newly graduated nurses are constantly exposed to occupational stressors that persist even during a pandemic, possibly maintaining a relatively stable pattern of burnout [13, 72]. Due to limited clinical experience, novice nurses are rarely assigned to perform demanding tasks independently. They reported that preceptorship, peer support, and teamwork strengthened emotional bonds in the workplace and alleviated EE during the pandemic [13]. Strengthened communication within clinical teams and closer connections with decision‐makers may have improved access to timely information and enhanced nurses’ sense of control over their work [73]. In addition, the public recognition healthcare professionals received during the pandemic, along with the opportunity to personally witness the direct value of their care to patients, may have enhanced their professional identity and sense of PA [73]. These potentially protective experiences may have partially counteracted the increased emotional demands brought on by the pandemic. Notably, some studies reported positive outcomes during this period, with new nurses rapidly developing stronger professional identity, higher clinical competence, and greater emotional maturity under intense pressure [13]. Although no statistically significant or clinically important impact of the pandemic was observed, it is essential to safeguard their mental health and provide ongoing support during public health emergencies to prevent long‐term negative consequences.

Our analysis identified regional variations in burnout, with newly graduated nurses in Asia reporting significantly higher DP scores and lower PA scores compared to their non‐Asian counterparts. This finding is consistent with previous meta‐analyses indicating that nurses in Asia experience the highest levels of burnout [16]. Regional differences were also identified in the meta‐analysis of physician burnout, indicating higher levels of burnout in Asia, and China had the highest burnout levels when grouped by country [4]. However, these findings should not be interpreted as definitive evidence of an Asia‐specific pattern. Only three studies were included in the non‐Asian subgroup, and the unequal distribution of studies between the Asian and non‐Asian subgroups may have limited the precision and stability of the subgroup estimates. In addition, the predominance of studies conducted in China means that the observed findings may partly reflect country‐specific healthcare contexts rather than regional differences alone. The observed regional variation may be related to a combination of health‐system, organizational, and sociocultural factors. The shortage of nurses is more severe in Asian countries, where the large and growing populations—particularly in China—place enormous pressure on healthcare systems, further exacerbating the imbalance in nurse‐to‐patient ratios [5, 16, 35]. Compared to Western countries, Asia’s economic conditions and healthcare systems are less developed, resulting in lower salaries and benefits for nurses in Asia, as well as less trust and respect in the workplace [16, 35]. Such structural and societal constraints exacerbate work stress and contribute to burnout among newly graduated nurses in Asia. Moreover, in collectivist cultures, particularly in many Asian societies, expressing emotional distress is often seen as a sign of weakness, and to maintain social harmony, people tend to suppress emotions, which affects mental and physical health [74]. Such cultural norms may also influence how burnout is experienced or reported. These results suggest that regional differences in burnout may be influenced by differences in cultural expectations, healthcare system structures, and organizational and governmental support for newly graduated nurses. These findings highlight the need to develop interventions and workforce policies tailored to the cultural contexts of different regions.

We identified various sociodemographic, personal traits and competence, work‐related, and other factors associated with burnout among newly graduated nurses. The main sociodemographic factors associated with burnout among newly graduated nurses are age, gender, educational level, and work tenure. Consistent with other studies, younger nurses showed higher burnout, possibly because they lacked work experience or other job resources, resulting in more stress [75]. Female nurses face greater family responsibilities and challenges in balancing work and family life, and thus a higher risk of burnout [2, 35]. With a higher educational level, nurses learn how to access and utilize resources, thereby safeguarding their mental health. On the other hand, higher education may exacerbate the perception of a gap between expectations and reality, leading to stress [38, 43, 76]. The relationship between work experience and burnout remains inconclusive, possibly due to the influence of other factors on burnout development, resulting in diverse patterns [9]. More longitudinal studies are needed to examine the development process of burnout from early career life. However, the evidence for sociodemographic factors was inconsistent across studies; therefore, these variables should not be interpreted as definitive factors associated with burnout among newly graduated nurses.

Regarding personal traits and competence, lack of professional competence, insufficient psychological capital, and utilizing negative coping strategies are associated with burnout. Newly graduated nurses experience a lack of preparedness for the job and a lack of confidence, knowledge, and skills to provide care, which can turn into stressors [77]. Enhanced professional competence can help newly graduated nurses cope with various situations at work and thus reduce the risk of burnout [35]. Psychological capital, such as self‐efficacy and resilience, also helps them through the transition and reduces burnout [41]. Individuals with high psychological resilience tend to adopt more proactive, problem‐solving coping strategies and seek social support to manage workplace stressors [43]. New nurses may experience burnout even before graduation; therefore, incorporating psychological capital development and avoiding passive coping strategies into nursing education can help prevent burnout when entering the workforce [76]. These findings emphasize the importance of improving competencies and developing personal inner resources of new nurses through teaching and training.

Work‐related factors such as quality of work life, transition shock, work environment, and workplace bullying and incivility are also associated with burnout. Poor quality of work life due to high workloads and work‐life conflicts can lead to low job satisfaction and burnout [75]. Incomplete adaptation to work, the mismatch between reality and expectations, and lack of social support can cause transition shock and negative emotions [43]. Despite the professional education received, sudden challenges in the actual clinical environment often exceed the expectations of newly graduated nurses, resulting in significant psychological stress. Creating an open and supportive work environment can greatly contribute to the well‐being of healthcare workers [70, 77]. Although many hospitals provide supportive environments through preceptorship, the lack of suitable preceptors and negative interactions with them in reality have led to undesirable outcomes [69, 77]. Negative behaviors such as bullying or violence are also reported by newly graduated nurses and have been shown to discourage them from approaching and asking for help from senior nurses [69, 77]. Healthcare organizations should achieve a balance between work demands and resources by increasing resource investment and improving the work environment.

Other factors, such as social support, are also essential. Newly graduated nurses with more social support can be helped with the transition to clinical practice and can alleviate burnout [21, 46]. Building multidimensional social networks with different sources of support, such as family, friends, preceptors, and colleagues, by talking and receiving emotional support, is very beneficial [70, 77]. Preceptors and colleagues can also provide advice, feedback, and assessment at work, which makes them feel supported and helps to enhance learning [77].

### 4.1. Implications for Nursing Management and Practice

The findings emphasize the importance of improving work competencies, reducing transition shocks, establishing a supportive work environment, and providing culturally sensitive support. During nursing education, programs such as coping skills training and resilience training should be established to reduce the risk of burnout [13, 76]. Nursing education programs may provide more practical opportunities or simulations using VR technology during education to prepare nursing students better for work [69]. Healthcare organizations can identify the specific needs and shortcomings of new nurses upon entry into the workforce, offering personalized career planning and support [78]. Healthcare organizations should provide structured orientation, accessible clinical supervision, and regular constructive feedback in the early transition‐to‐practice period [77]. During this period, healthcare organizations should regularly assess individual competencies and supervision feedback and gradually expand the scope of independent responsibilities rather than basing decisions strictly on length of tenure. This approach may help reduce the stress and loss of confidence associated with an abrupt transition from student status to independent clinical responsibility. The selection of preceptors should be based not only on seniority but also on their clinical competence in the relevant practice area, communication and feedback skills, willingness to support novice nurses, and capacity to offer consistent practical and emotional guidance. Adequate staffing and protected time are also needed to ensure that preceptors are not prevented by their own workload from providing effective supervision and feedback [77]. However, in institutions with limited staffing resources, hospitals can also adopt a flexible and scalable support approach, which includes designating a primary preceptor and establishing a small, trained backup preceptor team. Workplace bullying and incivility should be prevented by enhancing training for leaders and preceptors and promptly verifying and addressing any complaints. Improving workplace relationships and establishing support groups enable newly graduated nurses to access resources for seeking help and sharing experiences and needs [69, 78].

Geographic differences also show the impact of the healthcare system and cultural factors. In cultures where help‐seeking is not common, regular psychological assessments need to be provided to identify problems promptly and to provide free or affordable counseling. In many Asian countries where collectivism and hierarchical relationships are more pronounced, support systems that prioritize team‐based instruction and organizational harmony may prove more effective. Regions or countries with less developed healthcare systems should strive to ensure adequate staffing levels and enhance the welfare of new nurses.

In summary, the results underscore the need to shift the focus from passive responses after burnout has occurred to more proactive nursing management strategies. These include early risk identification, strengthening support during the transition period, and creating a work environment that enables newly graduated nurses to achieve safe and sustainable professional development.

### 4.2. Strengths and Limitations

The current study has several advantages. First, we followed the JBI guidelines and registered our review protocol in PROSPERO and also followed the PRISMA reporting guidelines to ensure a transparent, accurate, and standardized review process. Second, low‐quality studies were excluded from this study to ensure the quality of evidence and enhance reliability. Third, this systematic review quantified pooled scores for each dimension of burnout among newly graduated nurses through a meta‐analysis and synthesized a wide range of evidence on associated factors, providing stronger evidence than prior studies. Furthermore, we conducted subgroup analyses and sensitivity analyses to explore potential sources of heterogeneity and to ensure the robustness of the results. Therefore, the results of this systematic review and meta‐analysis offer evidence‐based support for nursing administrators in developing interventions to reduce burnout among newly graduated nurses.

This study also has several limitations. First, due to differences in sampling methods, data collection methods, study settings, and other factors, subgroup analyses and exploratory meta‐regression analyses were unable to explain significant heterogeneity, which may have affected the precision of the pooled estimates and warrants further investigation. Second, the regional subgroup comparisons were based on an unequal number of studies, and many of the included studies were conducted in China, which limits the precision and generalizability of our findings. Also, the cross‐regional comparison is incomplete, lacking data from America and Africa. Future research should consider conducting multicenter comparative studies across multiple regions to better understand the regional variations. Third, this study did not address the adequacy of using a single cutoff point across different countries and languages. Future research should examine measurement invariance across languages and cultures to accommodate different target populations and language contexts, thereby ensuring that observed differences do not arise from translation or scale adaptation, ultimately enhancing comparability and accuracy. Fourth, defining newly graduated nurses as nurses with up to 3 years of experience may have obscured temporal variations in burnout across the early‐career transition period. However, insufficient data for further disaggregation prevents us from conducting subgroup analyses to compare burnout among new nurses with varying lengths of experience. Finally, the cross‐sectional nature of most included studies restricts inferring causality. In summary, caution is required when drawing definitive conclusions in interpreting the results of the current study.

## 5. Conclusions

The meta‐analysis revealed a high level of burnout among newly graduated nurses, especially in Asia, suggesting a need for culturally sensitive support. Although burnout levels did not show statistically significant or clinically important differences before versus during the pandemic, continued attention to the well‐being of this population remains essential. The factors associated with burnout among newly graduated nurses are complex and require the development of evidence‐based interventions to improve the work competencies of this group, reduce transition shocks, and create supportive work environments to address burnout, ultimately reducing turnover and improving the quality of healthcare delivery. This review enhances the understanding of the severity and associated factors of burnout among newly graduated nurses and guides future intervention strategies and research design.

## Author Contributions

Yao Zhou: conceptualization, methodology, formal analysis, investigation, data curation, writing–original draft, and writing–review and editing. Zhexi Ying: methodology, formal analysis, investigation, data curation, and writing–review and editing. Akiko Kondo: methodology, supervision, and writing–review and editing.

## Funding

No funding was received for this study.

## Conflicts of Interest

The authors declare no conflicts of interest.

## Supporting Information

Additional supporting information can be found online in the Supporting Information section.

## Supporting information


**Supporting Information 1** Supporting File 1: Supporting Table S1: Search strategies and results; Supporting Table S2: Quality assessment of cross‐sectional studies; Supporting Table S3: Quality assessment of cohort studies; Supporting Table S4: Results of meta‐regression analyses; Supporting Table S5: Narrative synthesis of reported factors; Supporting Figure S1: Sensitivity analysis results.

**Supporting Information 2** Supporting File 2: PRISMA 2020 checklist.

## Data Availability

The data that support the findings of this study are available from the corresponding author upon reasonable request.
