# Supplementary material for: Burnout Levels and Associated Factors Among Newly Graduated Nurses: A Systematic Review and Meta‐Analysis
Source: J Nurs Manag. 2026 Aug 17;2026:4994782. doi: 10.1155/jonm/4994782 (PMC13479804; doi:10.1155/jonm/4994782)
Supplement: Supplementary file 2 [file JONM-2026-4994782-s002.docx]

**Supporting Table S1.** Search strategies and results.

| **Database** | **Search formula** | **Results** |
| --- | --- | --- |
| PubMed | ("newly graduate* nurse*" OR "new graduate* nurse*" OR "graduate* nurse*" OR "newly qualified nurse*" OR "novice nurse*" OR "fresh nurse*" OR "first year nurse*" OR "newly registered nurse*" OR "newly graduated registered nurse*" OR "new staff nurse*" OR "newly licensed nurse*" OR new nurse* OR "neophyte nurse*") AND ("Burnout, Professional"[Mesh] OR "Burnout, Psychological"[Mesh] OR Burnout) | 998 |
| CINAHL | ( “newly graduate* nurse*” OR “new graduate* nurse*” OR “graduate* nurse*” OR “newly qualified nurse*” OR “novice nurse*” OR “fresh nurse*” OR “first year nurse*” OR “newly registered nurse*” OR “newly graduated registered nurse*” OR “new staff nurse*” OR “newly licensed nurse*” OR new nurse* OR “neophyte nurse*” ) AND ( "Burnout, Professional"[Mesh] OR "Burnout, Psychological"[Mesh] OR Burnout ) | 378 |
| MEDLINE | ( “newly graduate* nurse*” OR “new graduate* nurse*” OR “graduate* nurse*” OR “newly qualified nurse*” OR “novice nurse*” OR “fresh nurse*” OR “first year nurse*” OR “newly registered nurse*” OR “newly graduated registered nurse*” OR “new staff nurse*” OR “newly licensed nurse*” OR new nurse* OR “neophyte nurse*” ) AND ( "Burnout, Professional"[Mesh] OR "Burnout, Psychological"[Mesh] OR Burnout ) | 250 |
| Web of Science | (ALL=(“newly graduate* nurse*” OR “new graduate* nurse*” OR “graduate* nurse*” OR “newly qualified nurse*” OR “novice nurse*” OR “fresh nurse*” OR “first year nurse*” OR “newly registered nurse*” OR “newly graduated registered nurse*” OR “new staff nurse*” OR “newly licensed nurse*” OR new nurse* OR “neophyte nurse*”)) AND ALL=("Burnout, Professional"[Mesh] OR "Burnout, Psychological"[Mesh] OR Burnout) | 1014 |
| Japan Medical Abstracts Society (JMAS) | (((新人看護職/TH or 新人看護師/AL) or (新人看護職/TH or 新卒看護師/AL)) and ((心理的燃え尽き/TH or バーンアウト/AL) or (心理的燃え尽き/TH or 燃え尽き症候群/AL))) | 129 |
| China National Knowledge Infrastructure (CNKI) | （篇关摘：新护士 + 新毕业护士 + 新入职护士 + 新人护士 + 新手护士 + 低年资护士（模糊））AND（篇关摘：倦怠 + 精疲力竭（模糊）） | 69 |

**Supporting Table S2.** Quality assessment results using JBI critical appraisal tool for analytical cross-sectional studies.

| **Study** | **Q1** | **Q2** | **Q3** | **Q4** | **Q5** | **Q6** | **Q7** | **Q8** | **Percentage of “Yes”** | **Quality level** |
| --- | --- | --- | --- | --- | --- | --- | --- | --- | --- | --- |
| Cao et al., 2021 | Y | Y | Y | Y | Y | U | Y | U | 75% | moderate |
| Catarelli et al., 2023 | Y | Y | U | Y | Y | N | U | U | 50% | moderate |
| Dwyer et al., 2019 | Y | Y | Y | U | Y | Y | Y | Y | 87.5% | good |
| Gong et al., 2022 | Y | Y | Y | Y | Y | Y | Y | Y | 100% | good |
| Guo et al., 2022 | Y | Y | Y | Y | Y | Y | Y | U | 87.5% | good |
| Hu and Xiao, 2022 | Y | Y | Y | Y | N | N | Y | U | 62.5% | moderate |
| Kaneko, 2012 | Y | Y | Y | Y | N | N | Y | U | 62.5% | moderate |
| Kim and Yang, 2023 | N | Y | U | Y | Y | Y | U | Y | 62.5% | moderate |
| Kim et al., 2019 | Y | Y | U | Y | Y | Y | U | U | 62.5% | moderate |
| Kim et al., 2022 | N | Y | Y | Y | Y | U | Y | U | 62.5% | moderate |
| Laschinger, 2011 | N | Y | Y | Y | N | N | Y | U | 50% | moderate |
| Laschinger and Grau, 2011 | N | Y | Y | Y | N | N | Y | U | 50% | moderate |
| Laschinger and Read, 2016 | N | Y | Y | Y | N | N | Y | U | 50% | moderate |
| Laschinger et al., 2010 | N | Y | Y | Y | N | N | Y | U | 50% | moderate |
| Laschinger et al., 2012a | N | Y | Y | Y | N | N | Y | U | 50% | moderate |
| Laschinger et al., 2012b | Y | Y | Y | Y | N | N | Y | U | 62.5% | moderate |
| Laschinger et al., 2015 | Y | N | Y | Y | N | N | Y | U | 50% | moderate |
| Ma et al., 2020 | Y | Y | Y | Y | N | N | Y | N | 62.5% | moderate |
| Ma et al., 2021 | Y | Y | U | Y | Y | Y | U | U | 62.5% | moderate |
| Ma et al., 2022 | Y | Y | Y | Y | N | N | Y | N | 62.5% | moderate |
| Molina-Mula et al., 2022 | N | Y | U | Y | Y | Y | Y | Y | 75% | moderate |
| Nakazawa et al., 2024 | N | Y | Y | U | Y | Y | Y | U | 62.5% | moderate |
| Peng et al., 2015 | Y | Y | U | Y | N | N | Y | U | 50% | moderate |
| Ren and Kim, 2023 | Y | Y | U | Y | Y | Y | U | Y | 75% | moderate |
| Serafin et al., 2022 | Y | Y | Y | U | N | N | Y | U | 50% | moderate |
| Shi et al., 2018 | Y | N | U | U | Y | Y | Y | U | 50% | moderate |
| Sun et al., 2021 | Y | Y | U | Y | Y | Y | U | U | 62.5% | moderate |
| Tan et al., 2021 | Y | Y | Y | Y | N | N | Y | U | 62.5% | moderate |
| Theodosius et al., 2020 | Y | Y | Y | Y | N | N | Y | U | 62.5% | moderate |
| **Study** | **Q1** | **Q2** | **Q3** | **Q4** | **Q5** | **Q6** | **Q7** | **Q8** | **Percentage of “Yes”** | **Quality level** |
| Wang et al., 2023 | Y | Y | Y | U | Y | Y | Y | U | 75% | moderate |
| Xie et al., 2020 | Y | Y | U | Y | Y | Y | Y | U | 75% | moderate |
| Xie et al., 2022 | Y | Y | Y | Y | N | N | Y | U | 62.5% | moderate |
| Xu et al., 2015 | Y | Y | Y | Y | Y | N | Y | U | 75% | moderate |
| Xu et al., 2022 | Y | Y | Y | Y | Y | Y | Y | Y | 100% | good |
| Zeng and Cui,2014 | Y | Y | Y | Y | N | N | Y | U | 62.5% | moderate |
| Zeng et al., 2023 | Y | Y | U | Y | Y | U | Y | U | 62.5% | moderate |
| Zhai et al., 2023 | Y | Y | Y | Y | N | N | Y | U | 62.5% | moderate |
| Zhao et al., 2023 | Y | Y | Y | Y | Y | Y | Y | Y | 100% | good |

Notes: Y: Yes; N: No; U: Unclear.

Based on the proportion of “yes” responses, the quality of studies was categorized as good (≥80%), moderate (50-79%), or poor (<50%).

Questions: Q1: Were the criteria for inclusion in the sample clearly defined?

Q2: Were the study subjects and the setting described in detail?

Q3: Was the exposure measured in a valid and reliable way?

Q4: Were objective, standard criteria used for measurement of the condition?

Q5: Were confounding factors identified?

Q6: Were strategies to deal with confounding factors stated?

Q7: Were the outcomes measured in a valid and reliable way?

Q8: Was appropriate statistical analysis used?

**Supporting Table S3.** Quality assessment results using JBI critical appraisal tool for cohort studies.

| **Study** | **Q1** | **Q2** | **Q3** | **Q4** | **Q5** | **Q6** | **Q7** | **Q8** | **Q9** | **Q10** | **Q11** | **Percentage of “Yes”** | **Quality level** |
| --- | --- | --- | --- | --- | --- | --- | --- | --- | --- | --- | --- | --- | --- |
| Chang and Kim, 2022 | Y | Y | Y | N | N | U | Y | Y | Y | N | U | 54.6% | moderate |
| Chen, 2012 | Y | Y | Y | N | N | N | Y | Y | Y | Y | U | 63.6% | moderate |
| Rudman et al., 2020 | Y | Y | U | Y | Y | U | U | Y | Y | Y | Y | 72.7% | moderate |
| Suzuki et al., 2006 | Y | Y | U | Y | Y | Y | Y | U | Y | Y | U | 72.7% | moderate |
| Suzuki et al., 2008 | Y | Y | U | Y | Y | Y | Y | Y | Y | Y | U | 81.8% | good |
| West et al., 2007 | Y | Y | Y | N | N | Y | Y | Y | N | N | N | 54.6% | moderate |

Notes: Y: Yes; N: No; U: Unclear.

Based on the proportion of “yes” responses, the quality of studies was categorized as good (≥80%), moderate (50-79%), or poor (<50%).

Questions: Q1: Were the two groups similar and recruited from the same population?

Q2: Were the exposures measured similarly to assign people to both exposed and unexposed groups?

Q3: Was the exposure measured in a valid and reliable way?

Q4: Were confounding factors identified?

Q5: Were strategies to deal with confounding factors stated?

Q6: Were the groups/participants free of the outcome at the start of the study (or at the moment of exposure)?

Q7: Were the outcomes measured in a valid and reliable way?

Q8: Was the follow up time reported and sufficient to be long enough for outcomes to occur?

Q9: Was follow up complete, and if not, were the reasons to loss to follow up described and explored?

Q10: Were strategies to address incomplete follow up utilized?

Q11: Was appropriate statistical analysis used?

**Supporting Table S4.** Results of the univariable meta-regression analyses.

| **Subscale** | **Moderator** | **n** | **β (95% CI)** | **P value** | **R² (%)** | **Residual I² (%)** | **QE (p-value)** |
| --- | --- | --- | --- | --- | --- | --- | --- |
| **EE** | Publication year | 12 | 0.096  (−0.460 to 0.652) | 0.708 | 0.0 | 98.66 | 913.43 (<0.001) |
|  | Sample size, per 100 participants | 12 | −0.21  (−0.61 to 0.19) | 0.268 | 5.27 | 97.93 | 583.17 (<0.001) |
|  | Female proportion, per 1 percentage point | 7 | −0.091  (−0.725 to 0.543) | 0.728 | 0.0 | 99.17 | 497.10 (<0.001) |
|  | Mean age, per year | 4 | 0.356  (−12.137 to 12.850) | 0.914 | 0.0 | 99.40 | 386.51 (<0.001) |
| **DP** | Publication year | 12 | 0.107  (−0.400 to 0.614) | 0.647 | 0.0 | 99.49 | 1867.43 (<0.001) |
|  | Sample size, per 100 participants | 12 | −0.15  (−0.54 to 0.25) | 0.428 | 0.0 | 99.35 | 1021.80 (<0.001) |
|  | Female proportion, per 1 percentage point | 7 | −0.008  (−0.630 to 0.615) | 0.976 | 0.0 | 99.76 | 1525.11 (<0.001) |
|  | Mean age, per year | 4 | −0.353  (−9.832 to 9.125) | 0.887 | 0.0 | 99.62 | 426.13 (<0.001) |
| **PA** | Publication year | 12 | −0.726  (−1.477 to 0.024) | 0.057 | 25.68 | 99.64 | 1598.86 (<0.001) |
|  | Sample size, per 100 participants | 12 | 0.02  (−0.73 to 0.76) | 0.964 | 0.0 | 99.68 | 776.69 (<0.001) |
|  | Female proportion, per 1 percentage point | 7 | −0.662  (−1.482 to 0.157) | 0.092 | 35.53 | 99.75 | 1996.40 (<0.001) |
|  | Mean age, per year | 4 | 2.496  (−2.366 to 7.359) | 0.158 | 57.07 | 97.33 | 83.32  (<0.001) |

Note: n indicates the number of studies included in each univariable meta-regression analysis. β represents the regression coefficient and indicates the expected change in the pooled outcome associated with a one-unit increase in the moderator. All tests were two-tailed, and P < 0.05 was considered statistically significant. R² represents the proportion of between-study heterogeneity explained by the moderator. Residual I² indicates the percentage of remaining variability attributable to between-study heterogeneity after inclusion of the moderator. QE is the test statistic for residual heterogeneity; a significant QE (p-value < 0.05) indicates that substantial unexplained heterogeneity remains.

**Supporting Table S5.** Narrative synthesis of reported factors associated with burnout among newly graduated nurses.

| **Category** | **Factors** | **Studies reporting a positive association** | **Studies reporting a negative association** | **Studies reporting no statistically significant association** |
| --- | --- | --- | --- | --- |
| **Sociodemographic factors** | **Age** |  | Dwyer et al., 2019; Ma et al., 2021; Xu et al., 2022; Zheng et al., 2023 | Cao et al., 2021; Catarelli et al., 2023; Nakazawa et al., 2024; Shi et al., 2018; Zhao et al., 2023; Zheng et al., 2023 |
|  | **Income** |  | Zheng et al., 2023 | Ma et al., 2021; Zhao et al., 2023 |
|  | **Female gender** | Xie et al., 2020; Xu et al., 2015; Wang et al., 2023 |  | Catarelli et al., 2023; Dwyer et al., 2019; Ma et al., 2021; Nakazawa et al., 2024; Shi et al., 2018; Zeng et al., 2023; Zhao et al., 2023 |
|  | **Educational level** | Cao et al., 2021; Nakazawa et al., 2024; Zhao et al., 2023 | Xu et al., 2022; Zheng et al., 2023 | Catarelli et al., 2023; Ma et al., 2021; Shi et al., 2018; Wang et al., 2023; Xie et al., 2021; Zeng et al., 2023 |
|  | **Unmarried status** | Ma et al., 2021; Zeng et al., 2023 | Xu et al., 2022 | Dwyer et al., 2019; Nakazawa et al., 2024; Xie et al., 2021; Zheng et al., 2023 |
|  | **Work tenure** | Kim et al., 2022; Wang et al., 2023 | Xu et al., 2022; Zhao et al., 2023; Zheng et al., 2023 | Shi et al., 2018 |
| **Personal traits and competence factors** | **Nurses' core competence** |  | Cao et al., 2021; Gong et al., 2022; Ma et al., 2021; Tan et al., 2023; Wang et al., 2023; Xie et al., 2020 |  |
|  | **Psychological capital** |  | Cao et al., 2021; Dwyer et al., 2019; Laschinger et al., 2015; Laschinger and Grau, 2011; Nakazawa et al., 2024; Shi et al., 2018; Tan et al., 2023; Xie et al., 2020; Xie et al., 2022; Zeng and Cui, 2014; Zhai et al., 2023; Zheng et al., 2023 |  |
|  | **Work engagement** |  | Nakazawa et al., 2024; Sun et al., 2021; Xie et al., 2020; Zhai et al., 2023 |  |
|  | **Psychological empowerment** |  | Guo et al., 2022; Ren and Kim, 2023 |  |
| **Category** | **Factors** | **Studies reporting a positive association** | **Studies reporting a negative association** | **Studies reporting no statistically significant association** |
| **Personal traits and competence factors** | **Positive and active coping strategies** |  | Cao et al., 2021; Chang and Kim, 2022; Kaneko, 2012; Nakazawa et al., 2024; Wang et al., 2023; West et al., 2007; Zhao et al., 2023 |  |
| **Work-related factors** | **Quality of work life** |  | Laschinger et al., 2015; Laschinger and Grau, 2011; Laschinger and Read, 2016; Nakazawa et al., 2024; Sun et al., 2021; Zeng et al., 2023 | Catarelli et al., 2023 |
|  | **Supportive work environment** |  | Dwyer et al., 2019; Gong et al., 2022; Kim and Yang, 2023; Laschinger et al., 2015; Laschinger et al., 2010; Laschinger and Read, 2016; Laschinger et al., 2012a; Laschinger et al., 2012b; Peng et al., 2015; Zhao et al., 2023 |  |
|  | **Structural empowerment** |  | Dwyer et al., 2019; Laschinger et al., 2010; Laschinger et al., 2012a |  |
|  | **Job satisfaction** |  | Cao et al., 2021; Catarelli et al., 2023; Laschinger, 2011; Laschinger et al., 2012b; Nakazawa et al., 2024; Suzuki et al., 2008; West et al., 2007; Xie et al., 2020; Zeng et al., 2023 |  |
|  | **Internship time** |  | Xu et al., 2022 |  |
|  | **Hospital level** |  | Xie et al., 2020 | Shi et al., 2018 |
|  | **Workplace bullying and incivility** | Chang and Kim, 2022; Laschinger et al., 2010; Laschinger and Grau, 2011; Laschinger and Read, 2016; Laschinger et al., 2012b; Ren and Kim, 2023; Serafin et al., 2022; Shi et al., 2018; Zeng et al., 2023 |  |  |
| **Category** | **Factors** | **Studies reporting a positive association** | **Studies reporting a negative association** | **Studies reporting no statistically significant association** |
| **Work-related factors** | **Occupational stress** | Cao et al., 2021; Kaneko, 2012; Xie et al., 2022; Xu et al., 2022; Zhao et al., 2023; Zheng et al., 2023 |  |  |
|  | **Transition shock** | Cao et al., 2021; Hu and Xiao, 2022; Xu et al., 2022 |  |  |
|  | **Working during COVID-19 pandemic** | Serafin et al., 2022 |  |  |
|  | **Night shifts** | Xu et al., 2022; Zeng et al., 2023 | Zheng et al., 2023 |  |
| **Other factors** | **Social support** |  | Gong et al., 2022; Hu and Xiao, 2022; Nakazawa et al., 2024; Suzuki et al., 2006; Suzuki et al., 2008; Zhao et al., 2023 |  |
|  | **Sleep duration** |  | Zeng et al., 2023 |  |
|  | **Absence of social group membership** | Kim et al., 2022 |  |  |
|  | **Mobile phone addiction** | Ma et al., 2020; Ma et al., 2021 |  |  |

**Supporting****Figure S1.** Sensitivity analysis results by subscale: (a) emotional exhaustion; (b) depersonalization; (c) personal accomplishment.
